# *BRAF*-mutant hematopoietic malignancies

**DOI:** 10.18632/oncotarget.2448

**Published:** 2014-09-06

**Authors:** Omar Abdel-Wahab, Christopher Y. Park

**Affiliations:** Human Oncology and Pathogenesis Program, Leukemia Service, Dept. of Medicine, Memorial Sloan Kettering Cancer Center, New York, NY; Human Oncology and Pathogenesis Program, Dept. of Pathology, Dept. of Medicine, Memorial Sloan Kettering Cancer Center, New York, NY

Mutations activating the serine-threonine kinase *BRAF* have been identified in a variety of cancers since they were first described in 2002. Currently, it is estimated that *BRAF* is mutated in ~8% of all cancers [1]. Sequencing studies performed in a broad spectrum of hematologic malignancies have revealed that mutations in *BRAF*, although generally quite rare amongst leukemias and lymphomas, are strikingly enriched in 2 rare hematologic malignancies: hairy cell leukemia (HCL) and the systemic histiocytoses, Langerhans Cell Histiocytosis (LCH) and Erdheim-Chester Disease (ECD) (Figure).

Although the discovery of the high frequency *BRAF*V600E mutations in these conditions has encouraged the intiation of novel therapeutic strategies targeting *BRAF*V600E, they have also provided unexpected biological insights through investigation of the functional consequence of *BRAF*V600E. One issue addressed is the cell-of-origin in HCL and the histiocytoses, a debate that had remained unresolved for decades. While LCH has been proposed to arise from epidermal Langerhans cells or immature myeloid dendritic cell (DC) precursors, HCL has largely been considered to be a mature B cell malignancy, despite the fact that their immunophenotype and morphology are distinct from any known normal B cell population.

The recent discovery of *BRAF*V600E mutations in nearly 100% of HCL patients and 40-60% of LCH patients led our group [2; 3] and a group led by Drs. Miriam Merad and Carl Allen [4] to investigate the cell-of-origin in HCL and LCH by identifying cell populations in which the *BRAF*V600E mutation could be found. Our studies showed that the *BRAF*V600E mutation is present in highly purified long-term hematopoietic stem cells (LT-HSCs; lineage-negative CD34+ CD38- CD90+ CD45RA- cells) from HCL patients. Moreover, xenotransplantation of purified LT-HSCs from *BRAF*V600E mutant HCL patients gave rise to stable human grafts that propagated the *BRAF*V600E mutation. Berres *et al*. similarly identified the *BRAF*V600E mutation in CD34+ cells from LCH patients with multisystem disease by sequencing of purified cells as well as colonies derived from CD34+ cells from LCH patients grown in methycellulose (Figure).

The presence of the *BRAF*V600E mutation in hematopoietic stem/progenitor cells (HSPCs) of patients with seemingly disparate clinical disorders raises the question of the importance of such mutations in HSPCs. In the case of LCH, Berres *et al*. posited a disease pathogenesis model in which the cell-of-origin of the *BRAF*V600E mutation defines the extent and severity of the clinical disease (Figure). Thus, delineation of the precise HSPC that the *BRAF*V600E mutation arises in may provide further insights into how LCH relates to HCL. Similar studies of the related, but clinically distinct, histiocytic disorder ECD may be likewise enlightening. Functional evidence of whether *BRAF*V600E mutant CD34+ cells from LCH patients are capable of self-renewal *in vivo* in xenograft assays may also provide further insights about the HSPC compartment of these disorders.

**Figure 1:**
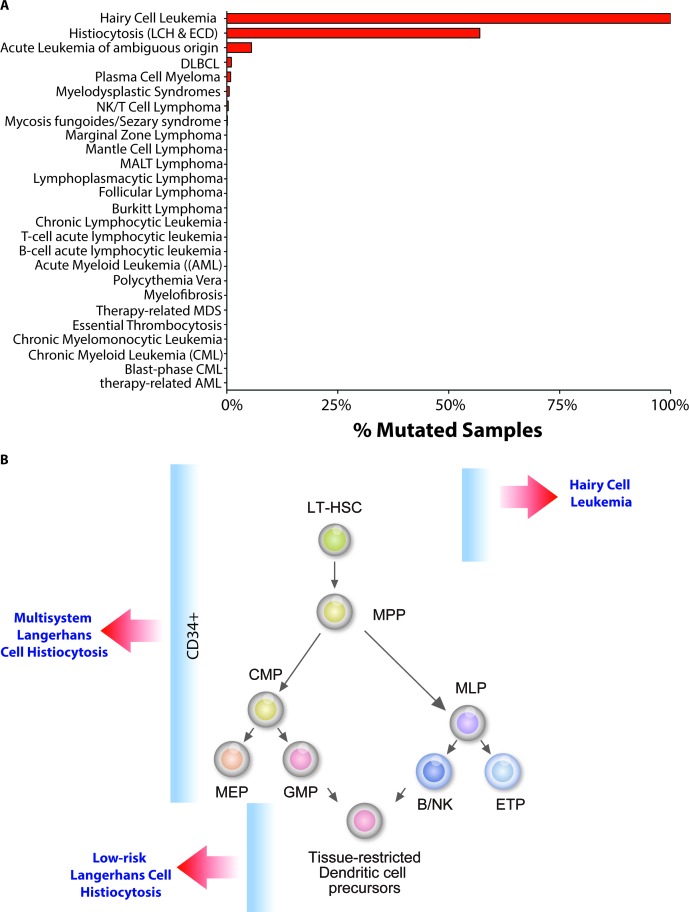
Spectrum and cell-of-origin of BRAFV600E-mutant hematopoietic disorders (A) Histogram of *BRAF*V600E mutations amongst hematopoietic malignancies (based on COSMIC search in July 2014). (B) Diagram of our current understanding of *BRAF*V600E mutation occurrences within the hierarchy of hematopoiesis in patients with hairy cell leukemia (HCL) and Langerhans Cell Histiocytosis (LCH).

Intriguingly, the presence of *BRAF*V600E mutations in HSPCs and the neoplastic cell proliferations in HCL and LCH is more than simple coincidence, as phenotypes induced by oncogenic BRAF depend on when they are expressed during hematopoiesis. Evaluation of the first *BRaf*V600E conditional knockin mouse model generated by Mercer *et al*. revealed that pan-hematopoietic expression of *BRaf*V600E from the endogenous *BRaf* locus resulted in a lethal, multi-system histiocytic disorder [5]. Using this model, we showed that expression of *BRaf*V600E in the earliest B cells resulted in increased self-renewal of early B-lineage cells as well as a number of other phenotypic hallmarks of human HCL disease [2]. In contrast, expression of *BRaf*V600E restricted to committed B cells failed to recapitulate numerous aspects of HCL observed in the pan-hematopoietic model (notably, phenotypic hairy cells were not seen in any model). Berres *et al*. observed a similar developmental-stage specific effect of *BRaf*V600E expression, as expression of *BRaf*V600E restricted to tissue-resident DCs failed to produce a multisystem histiocytic disorder, while expression of *BRaf*V600E in committed DC progenitors did [4].

Now that mutational and functional analyses have definitively demonstrated that specific *BRAF*V600E-harboring cells give rise to these conditions, further use of primary patient cells and mouse models will be necessary to determine how oncogenic *BRAF* induces specific gene expression programs in different cell types to induce disease phenotypes. Moreover, it will be interesting to compare and contrast these results by comparing transcriptional targets of BRAFV600E in solid tumors to determine the tissue-specific specific BRAFV600E transcriptional dependencies.

The identification of highly recurrent *BRAF*V600E mutations in HCL, LCH and ECD has stimulated a number of genomic efforts aimed at identifying what genetic alterations may be present in the non-*BRAF*V600E subset of patients. Here again, quite surprisingly, a related peculiar finding arose: the discovery of frequent *MAP2K1* mutations in *BRAF*V600E-wildtype patients with LCH [6] and 2 rare subtypes of HCL (the HCL variant and IGHV4-34-expressing HCL) [7]. Since ERK activation had been described previously in nearly all HCL and LCH/ECD patients, the discovery of another genetic alteration activating the MAP kinase pathway is perhaps not surprising. However, given the rarity of *MAP2K1* mutations in hematological malignancies as a group, the unexpected genetic parallels between the systemic histiocytoses and HCL are striking. We look forward to the novel insights gained from future studies in these disorders.
